# Shift in the seasonality of ixodid ticks after a warm winter in an urban habitat with notes on morphotypes of *Ixodes ricinus* and data in support of cryptic species within *Ixodes frontalis*

**DOI:** 10.1007/s10493-022-00756-1

**Published:** 2022-10-25

**Authors:** Ciara Reynolds, Jenő Kontschán, Nóra Takács, Norbert Solymosi, Attila D. Sándor, Gergő Keve, Sándor Hornok

**Affiliations:** 1 Department of Parasitology and Zoology, University of Veterinary Medicine, Budapest, Hungary; 2 Plant Protection Institute, Centre for Agricultural Research, ELKH, Budapest, Hungary; 3 ELKH-ÁTE Climate Change: New Blood-Sucking Parasites and Vector-Borne Pathogens Research Group, Budapest, Hungary; 4 Centre for Bioinformatics, University of Veterinary Medicine, Budapest, Hungary; 5 Department of Parasitology and Parasitic Diseases, University of Agricultural Sciences and Veterinary Medicine, Cluj-Napoca, Romania

**Keywords:** Phenology, *Cox*1 gene, 16S rRNA gene, Haplogroup, Morphological anomalies

## Abstract

This study was initiated to assess the seasonality and to investigate the morphology of questing ixodid ticks in an urban habitat in Central Europe, Hungary. A neglected part of a large cemetery, with sparse tree covering and dense lower vegetation, was sampled monthly from February 2019 to May 2021. All ticks were analyzed morphologically, and selected specimens by amplifying and sequencing two genetic markers. During the study 3818 ticks were collected, including *Ixodes ricinus* (n = 2772), *Ixodes frontalis* (n = 350) and *Haemaphysalis concinna* (n = 696). *Ixodes ricinus* adults and nymphs showed year-round activity, whereas *H. concinna* was not active during winter months and early spring. Most *I. frontalis* nymphs were collected in late winter and early spring, whereas the peak activity of larvae was during late autumn. Interestingly, during the spring, the peak activity of *I. ricinus* adults and nymphs was later (in May) when preceded by a warm winter in 2020. In contrast, the 2019 and 2021 spring activity peaks occurred in March and April after sharply rising temperatures in February. This shift in the peak activity of *I. ricinus* coincided with the initiation of questing activity of *H. concinna*. Three notably different morphotypes and four malformed specimens of *I. ricinus* were found. However, these were not significantly different in their mitochondrial haplotypes and phylogenetic clustering from typical specimens of this species. On the other hand, *I. frontalis* was represented by two remarkably different haplogroups, between which in the nymph stage there were no recognizable morphological differences, suggesting the status of these as cryptic species.

**Supplementary Information:**

The online version contains supplementary material available at 10.1007/s10493-022-00756-1.

## Introduction

In the era of globalization, by 2050 nearly 70% of human population will live in cities (UN DESA 2018). The surface covered by urban areas is steadily increasing worldwide, and these expand into natural habitats of wildlife. At the same time, urbanized regions include artificially maintained green surfaces, forming habitats in a mosaic-like arrangement (Dautel and Kahl 1999). This may be significant to consider from an epidemiological point of view, particularly in the context of pathogen reservoirs and transmitters (vectors).

Hard ticks (Acari: Ixodida, Ixodidae) are haematophagous ectoparasites of terrestrial vertebrates, with outstanding veterinary-medical importance as vectors in the temperate climate zone (Jongejan and Uilenberg 2004), the latter exemplified by Central Europe including Hungary. Previously, in the capital city of Hungary various urban habitats (forests, parks, cemeteries) were surveyed for the presence of ticks and tick-borne pathogens (Hornok et al. 2014). Neglected parts of cemeteries were found to be especially suitable to maintain large tick populations, most likely because of the restricted entry or near absence of medium-sized carnivores, entailing high population densities of small mammals and birds (Lussenhop 1977). However, although the year-round activity of ixodid ticks has previously been surveyed in the country (Hornok 2009), this later extensive collection of urban ticks focused on the spring tick season (Hornok et al. 2014), and in this way missed those tick species which were reported to have autumn and winter activity in Western Europe, particularly *Ixodes frontalis* (Agoulon et al. 2019; Plantard et al. 2021). Importantly, the year-round seasonality of *I. frontalis* has not been reported in Central Europe, but relevant data are available from surveys in Germany (Drehmann et al. 2019; Hauck et al. 2020).

This study was initiated primarily (1) to contribute to data on the complete seasonal activity of tick species occurring in urban biotopes in Central Europe, with special emphasis on *I. frontalis*. In addition, it was within the scope of this work to examine the detailed morphology of collected *Ixodes* specimens with two purposes: (2) to evaluate the occurrence of morphotypes within *I. ricinus* as reported for *Pholeoixodes* species (Hornok et al. 2017) and (3) to investigate any morphological differences between the 2 well-established mitochondrial lineages of *I. frontalis* which were discovered in Hungary and have sequence divergence exceeding that between closely related species (Hornok et al. 2016).

## Methods

### Sample collection

The tick collection site was chosen based on the results of a large-scale survey of urban biotopes in Budapest (Hornok et al. 2014). The exact location (coordinate) is not shown, in order to ensure long term undisturbed state of the relevant tick habitat for monitoring. This biotope is part of a large cemetery, where neglected parts had dense lower vegetation [grass, weed and nearly continuous ivy (*Hedera* sp.) covering] and sparse distribution of bushes and trees (Fig S1). This site was visited at a monthly interval, at the end of each month between February 2019 and May 2021 (i.e., for 28 months). Tick collections were performed under similar (dry) weather conditions and at the same time of the day. Ticks were collected from the vegetation by the dragging-flagging method, i.e., a white towel, measuring 1 × 1 m, was drawn over the vegetation for 1 h and checked every 10 s. During this the same 5, approx. 60 m long parallel transects were sampled regularly (i.e., 300 m^2^). Ticks attached to and removed from the collecting device were immediately put into and stored in 96% ethanol. Species were identified by using standard morphological keys (Estrada-Peña et al. 2017). Pictures were made and measurements were performed with a VHX-5000 digital microscope (Keyence, Osaka, Japan).

### Molecular analyses

DNA was extracted individually from two legs of ticks selected for morphological analysis. Ticks were disinfected on their surface with sequential washing for 15 s in 10% NaClO, in tap water and in distilled water. DNA was extracted with the QIAamp DNA Mini Kit (QIAGEN, Hilden, Germany) following the manufacturer's instruction, including an overnight digestion in tissue lysis buffer and proteinase-K at 56 °C. A negative control (tissue lysis buffer) was also processed in each set of tick samples, in order to monitor cross-contamination.

The 16S rRNA gene was chosen for the molecular-phylogenetic analyses of *Ixodes ricinus* (n = 24: 18 females, five males and one nymph) and *Ixodes frontalis* (n = 25: one female, 15 nymphs and nine larvae) including specimens selected for detailed morphological examination, because this target gene has a broad spectrum of corresponding sequences available for comparison from other studies in GenBank (e.g., Noureddine et al. 2011; Hornok et al. 2016). The method used in this study amplifies an approx. 460-bp-long fragment of the 16S rRNA gene of Ixodidae (Black and Piesman 1994) with the primers 16S + 1 (5′-CTG CTC AAT GAT TTT TTA AAT TGC TGT GG-3′) and 16S-1 (5′-CCG GTC TGA ACT CAG ATC AAG T-3′) (Integrated DNA Technologies, Leuven, Belgium) as reported (Hornok et al. 2016).

The cytochrome *c* oxidase subunit I (*cox*1) gene was chosen for the confirmation of *I. frontalis* haplotypes (A or B). A 710-bp-long fragment of this gene was amplified with a conventional PCR using the primers LCO1490 (forward: 5′-GGT CAA CAA ATC ATA AAG ATA TTG G-3′) and HCO2198 (reverse: 5′-TAA ACT TCA GGG TGA CCA AAA AAT CA-3′) (IDT) (Folmer et al. 1994) as reported (Hornok et al. 2016). All PCRs were run with sequence-verified positive control and negative control (non-template reaction mixture). Extraction controls and negative controls remained always PCR negative.

### Sequencing and phylogenetic analyses

Purification and sequencing of the PCR products were done by Biomi (Gödöllő, Hungary). Quality control and trimming of sequences were performed with BioEdit program, then alignment with GenBank sequences online by BLASTN (https://blast.ncbi.nlm.nih.gov). Sequences obtained in this study have been submitted to GenBank (accession numbers: OM108450-OM108465 for *I. ricinus* 16S rRNA gene, OM108447-OM108449 for *I. frontalis* 16S rRNA gene and OM108437-OM108443 for *I. frontalis cox*1 gene). Sequences from other studies (retrieved from GenBank) included in the phylogenetic analyses had 99–100% coverage with sequences from this study. This dataset was resampled 1000 × to generate bootstrap values. Phylogenetic analyses were conducted with the Neighbor-Joining method and p-distance model by using MEGA v.7.0.

### Statistical analyses and presentation of data

Tick abundance data were compared by Fisher’s exact test (α = 0.05). Tick activity was calculated from the monthly ratio of ticks, expressed as a percentage of all conspecific ticks of the same developmental stage collected in the relevant biotope during 1 year (2019, 2020) or during 5 months (2021) (Table S1). Quarterly and monthly tick numbers were also compared with Mood’s median test according to years (Fig S2). Data of daily mean temperatures were used to illustrate the background role of winter weather in the questing activity of ticks, because (1) these were available continuously on a daily basis, and (2) temperature data provided by meteorological observatories were shown to be strongly correlated with local, near-ground temperature measurements and thus tick activity (Daniel et al. 2015). The graphs showing daily mean temperature during winter months are available from the Hungarian Meteorological Service (OMSZ 2021).

## Results

### Species and seasonality of ticks

**Fig. 1:**
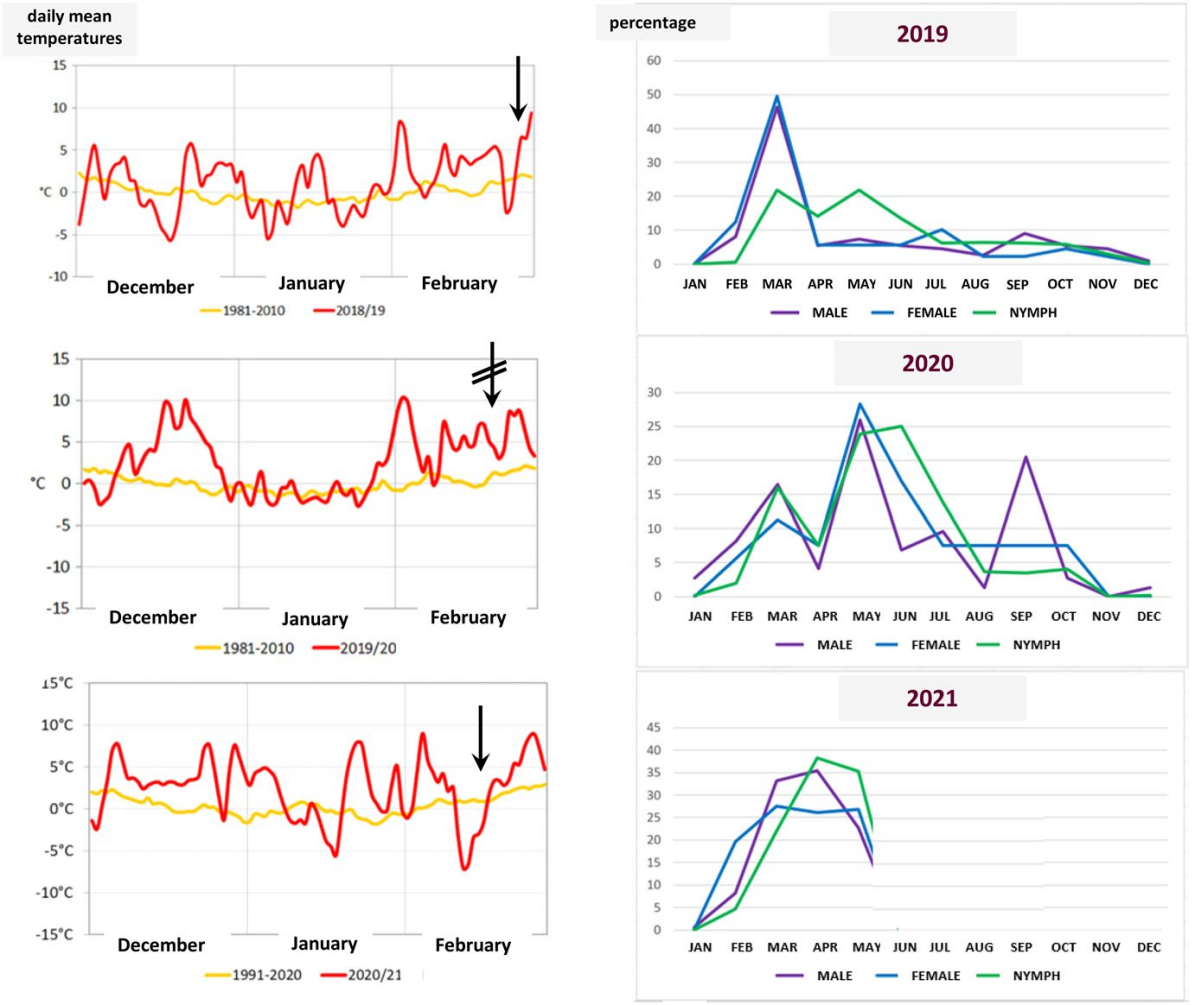
Left: mean daily winter temperatures (data from Hungarian Meteorological Service). Arrows indicate sharp rise in winter temperature, and crossed arrow the absence of this phenomenon. Right: activity curves of males, females and nymphs of *Ixodes ricinus* according to months shown below the diagram abbreviated

In the study period 3818 ticks were collected, including *I. ricinus* (n = 2772), *I. frontalis* (n = 350) and *Haemaphysalis concinna* (n = 696). Questing *I. ricinus* adults were present on the vegetation throughout the year (Fig. 1, Table S1). Males and females showed their peak activity in March in 2019, with a smaller peak of males in September and of females in July, whereas the spring peak of adults was later (in May) in 2020, and again earlier (in March–April) in 2021 (Fig. 1). Nymphs of this species also showed year-round questing activity, reaching the highest level in the spring (March and May) in 2019, and, similarly to the adults, later (in June) in 2020, and earlier (in April) in 2021 (Fig. 1). Larvae of *I. ricinus* were collected in peak numbers in May, in July and in May in 2019, 2020 and 2021, respectively. This is in line with quarterly cumulative tick numbers for all stages/sexes of *I. ricinus*: significantly higher number of ticks were collected from the annual tick population in the spring in 2019 than in 2020 (P < 0.001). Using the median of the sample sets (data smoothing) this was reflected by a flattening of the activity curve in 2020, compared to 2019 (Fig S2).

No activity of *H. concinna* was observed during winter months. This species initiated its questing activity in March of 2019, in May of 2020, and in April of 2021. The activity period was longer (9 months) in 2019 than in 2020 (6 months) (Table S1).

A single *I. frontalis* female was found in March, 2019. Nymphs of this species were collected in March (n = 3), but not in the autumn during 2019, then (after an absence in the spring) in September of 2020 (n = 2), followed by activity in February, March and April of 2021 (n = 10, 4, and 1, respectively). Larvae of *I. frontalis* were collected in October (n = 111) and November (n = 69) in 2019, then in October 2020 (n = 136), finally in March and April, 2021 (n = 10 and 3, respectively). This means that while the larvae were significantly associated with the autumn, nymphs predominated in the spring (Fisher exact test: P < 0.0001). In summary, *I. frontalis* was not found questing on the vegetation during late spring and summer months.

### Molecular and phylogenetic analysis of *Ixodes ricinus* morphotypes

**Fig. 2:**
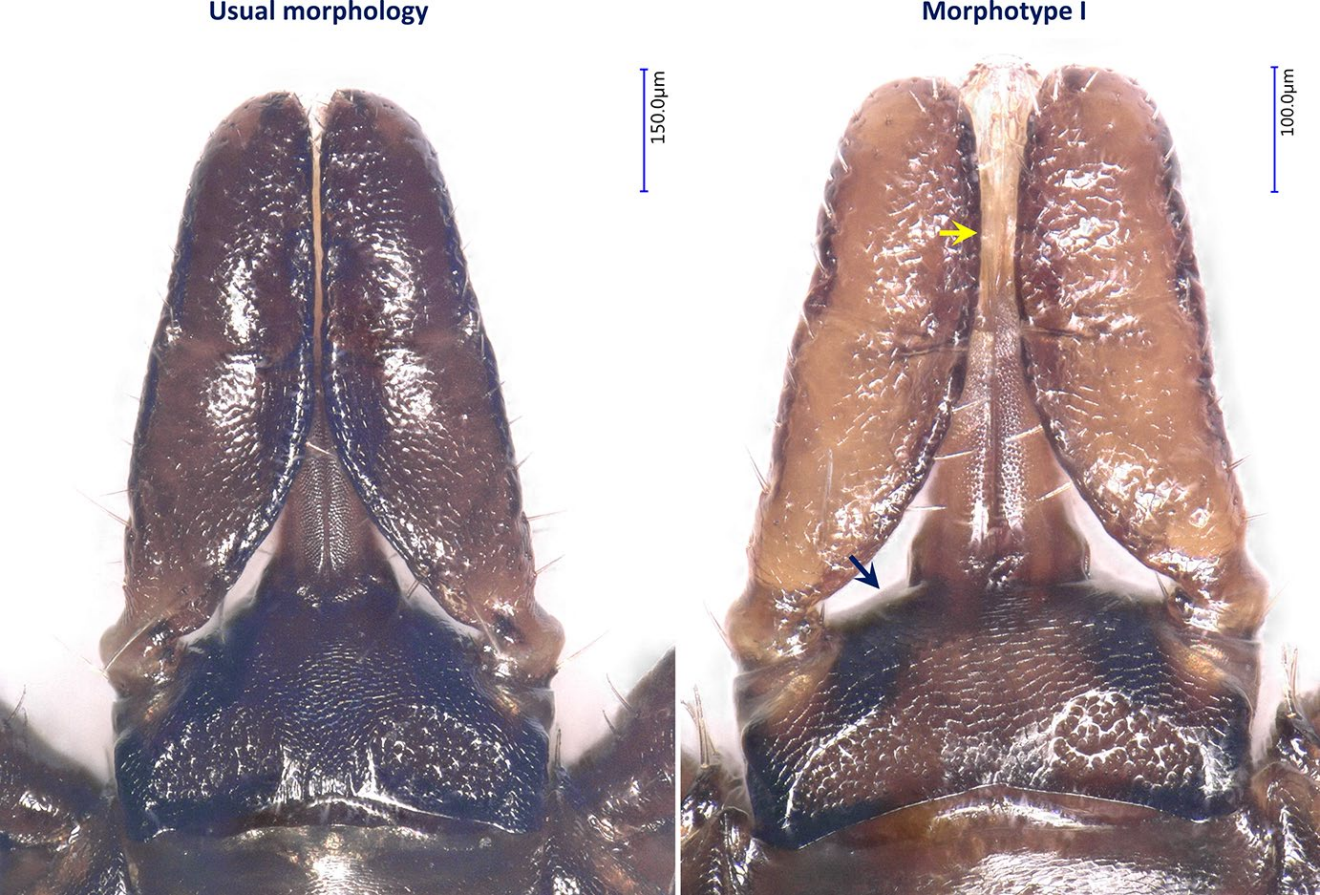
Gnathosoma of a ‘typical’ *Ixodes ricinus* female in comparison with that of morphotype I, both from the study material. The yellow arrow indicates medially curved palpal article III, and the dark blue arrow shows the flattened front of basis capituli around the hypostome

**Fig. 3:**
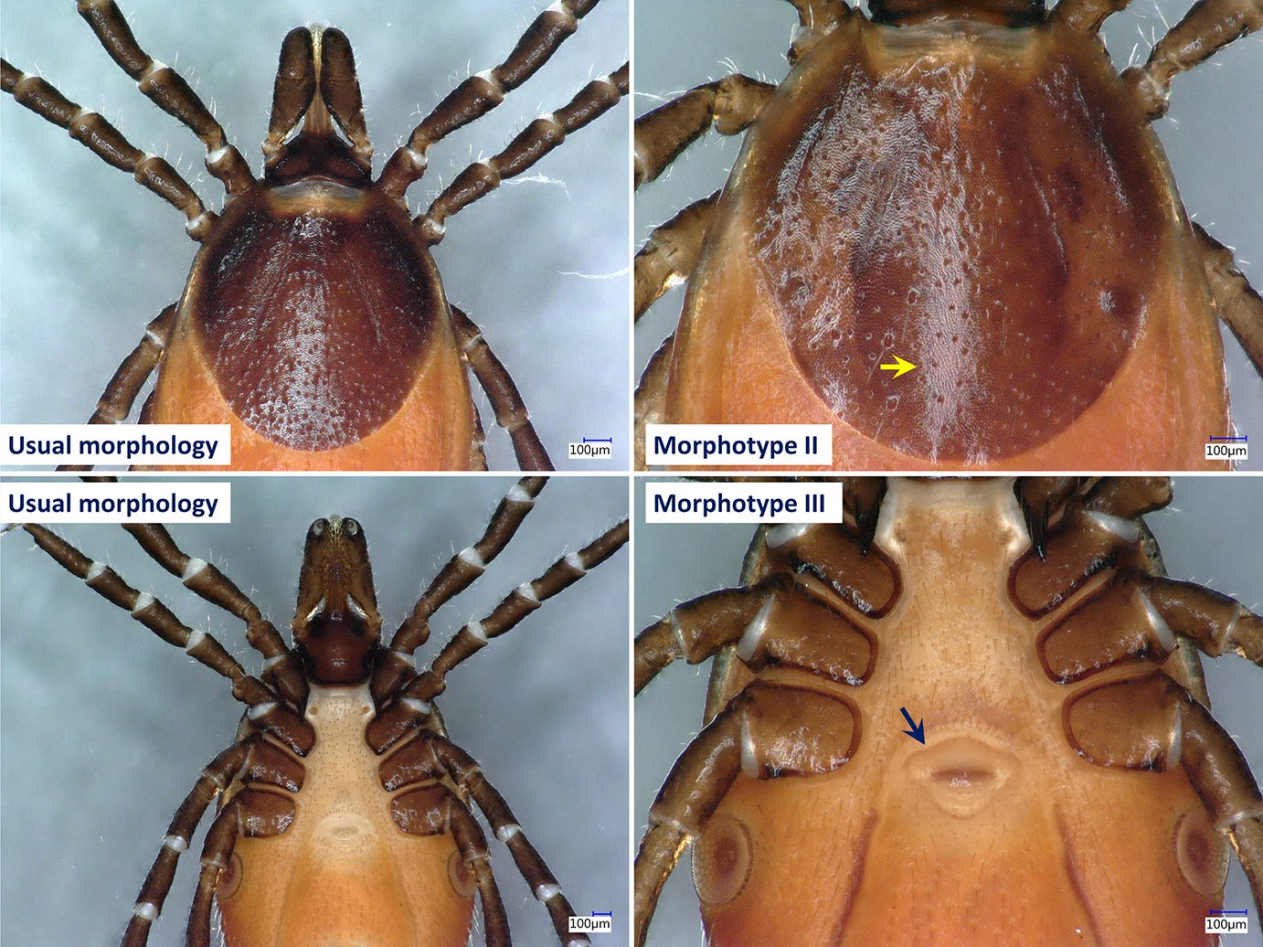
Distinguishing characters of morphotypes II–III from ‘typical’ females of *Ixodes ricinus*. The yellow arrow indicates smoother scutum with more scattered small pores, and the dark blue arrow shows the wavy edge of the genital pore

All specimens of *I. ricinus* collected during the study period that showed significant, unusual morphological character(s) were analyzed by sequence alignment and phylogenetic comparison of their 16S rRNA gene with ticks showing ‘usual morphology’ characteristic of this species. The ticks examined in this context included 3 unusual morphotypes of females: morphotype I (with flattened front of basis capituli around the hypostome making the head more rectangular than pentagonal, with medially curved palpal article III: Fig. 2, Fig S3), morphotype II (smoother scutum, with more scattered small pores: Fig. 3) and morphotype III (with wavy edge of the genital pore: Fig. 3). In addition, the following morphological anomalies were noted: deformity of the scutum in a female (Fig S4a); absence of groove separating adanal shields in a male (Fig S4b) and circumanal groove in a male (Fig S4c) and a nymph (Fig S4d).

Molecular analyses of the 16S rRNA gene showed that the above morphotypes and malformed specimens of *I. ricinus* were not more different from typical representatives of this species than from each other, or the ticks with ‘usual morphology’ from each other. In addition, these unusual specimens did not show consistent phylogenetic clustering (Fig S5).

### Molecular and morphological comparison of *Ixodes frontalis* haplogroups A and B

As confirmed by both *cox*1 (n = 24) and 16S rRNA gene (n = 25) analyses, *I. frontalis* haplotypes from both groups (A and B) were found in the cemetery but based on the molecularly analyzed 25 ticks haplogroup A (n = 19) predominated over B (n = 6). There was no overt difference between the seasonality of molecularly analyzed specimens, although among the seven larvae collected in October and November only haplotype-A was found (Table S2).

Six *I. frontalis* nymphs were selected for detailed morphological analysis: three from haplogroup A and three from haplogroup B. The *cox*1 sequence of three nymphs representing haplotype-A had 99.8–100% (653–654/654 bp) sequence identity with genotype A-Hu1 (KU170492) reported previously in Hungary. The *cox*1 sequence of three nymphs representing haplotype-B had 99.5–100% (651–654/654 bp) sequence identity with genotype B-Hu1 (KU170501) also reported in Hungary. Thus, the *cox*1 sequence divergence between nymphs from haplogroup A and B was approx. 8.6% (56/654 bp). This was confirmed by analysis of the 16S rRNA gene: the sequence identity between haplogroups A and B was only 96.3–96.5% (360–361/374 bp), amounting to a difference of 3.7%. Importantly, based on the *cox*1 gene, all five molecularly analyzed nymphs of haplogroup B had different sequences (GenBank: OM108437-OM108441), whereas 18 of 19 larvae and nymphs representing haplogroup A had identical sequences (OM108443). All molecularly analyzed *I. frontalis* larvae (seven from the autumn, two from the spring) belonged to haplogroup A.

The following species-specific morphological characters were observed to be uniform between the above six specimens. The idiosoma was lightly colored (compared to nymphs of *I. ricinus*), and the spiracle openings were dark brown. The scutum was slightly broader than long (width to length ratio was in the range of 1.005–1.05), posteriorly with scattered punctuations (Fig S6, blue arrow). Ventrally, the coxae had prominent external spur, and the anal groove was converging behind the anus (Fig S6, yellow arrows). On the gnathosoma, ‘frontal bumps’ were present, and auriculae had narrower anterior and broader posterior divisions (Fig S7, yellow arrows). The hypostome was pointed, the dental formula was 3/3 until mid-length (Fig S6, blue arrow). Anterior median scutal setae (sc1–3) were approx. 2 × longer (30–35 μm) than posterior scutal setae (sc4–5: 15–20 μm) (Fig S8). Length of lateral alloscutal setae anteriorly 180–200 μm (Fig S8, star). Spiracle opening was round, with 2–3 rows of regularly arranged large goblets (inner circle: approx 10, outer: approx. 20) (Fig S7, yellow arrow).

In summary, the morphological characters listed above did not show definable difference between nymphs of haplogroups A and B of *I. frontalis*.

## Discussion

In this study, the long-term seasonality and the haplotype-related morphology of ixodid ticks were assessed in an urban biotope. The results include the first evidence on the presence of questing *I. frontalis* on the vegetation in Hungary. The most likely explanation why this species was formerly not found in the same habitat (cemetery: Hornok et al. 2014) is that the previous study focused on the spring tick season, whereas here it was demonstrated that *I. frontalis* larvae have their activity peak late autumn. In addition, even during the present study only a single *I. frontalis* female was found and nymphs of this species could not be collected in the spring of 2020.

Regarding the seasonality of ixodid ticks, the year-round activity of *I. ricinus* males and females in the cemetery is not surprising in light of the fact that even in natural habitats adults of this tick species were found questing in each month of the year (Hornok 2009). However, in contrast to the urban habitat evaluated in this study, in natural habitats of Hungary the nymphs of this tick species did not show questing activity in most parts of the winter (Hornok 2009). The nearly continuous presence of *I. ricinus* nymphs questing on the vegetation in late autumn and in the winter of 2019/2020 (including November, December and January) may in part be due to the warmest winter of meteorological records in Europe during the relevant months (C3S 2020), and in Hungary the 3rd mildest winter (Fig. 1, data from the Hungarian Meteorological Service). Similarly increased activity of *I. ricinus* during the winter of 2019/2020 was reported in Switzerland (situated on the same latitude as Hungary) (Hornok et al. 2022).

In the urban habitat, all life cycle stages of *I. ricinus* had 1–2 months earlier activity peaks in 2019 and in 2021 than in 2020. Based on our data the peak questing activity appeared to be synchronized between developmental stages, thus bi-annual return to the former activity pattern is probably not due to the 2–3 years during which *I. ricinus* usually completes its development under a temperate climate (Randolph et al. 2002). In addition, although host availability is known to influence the temporal dynamics of *I. ricinus* (Cayol et al. 2017), similar temporal questing patterns of adults and nymphs (while having different host preferences) argues against a substantial role of host number fluctuations in the observed phenomenon of annual shifts in the peak activity of ticks. Rather, abiotic factors, i.e., daily temperatures during the winter preceding the spring tick season may explain this phenomenon: the continuously warm winter at the onset of 2020, in contrast to sharp increase of temperatures in February of 2019 and 2021 (Fig. 1, left panel). It is known that rapid fall in temperatures causes more *I. ricinus* developmental stages to suspend their activity (Randolph et al. 2000), and the opposite was reported to be relevant in the context of spring tick activity, i.e., extreme temperature rise preceding the spring tick season (as also experienced in 2019 and 2021: Fig. 1, left panel) may have promoted earlier spring activity peak of tick species in relevant years, as reported, explained earlier in the country-wide survey (Hornok 2009). In Central Europe, the increase in extreme temperature values is considerably higher than the corresponding average winter warming, and approximately 2.5 × higher than average global warming (Lorenz et al. 2019). Therefore, we hypothesize that extreme local warming (exceeding 10 °C in the course of a few days: Fig. 1, arrows) during February triggered earlier activity peak of *I. ricinus* (all stages) in 2019 and in 2021, but this was neutralized by the permanently warm winter at the beginning of 2020 (C3S 2020), causing later peaks and ‘flattened activity curve’. This was also confirmed by the significantly higher ratio of ticks questing earlier after a sharp temperature rise in February, in comparison with a spring tick season with less ticks after a permanently warm winter.

In case of *H. concinna*, adult questing activity started in the same months in urban as in natural habitats (April–May: Hornok 2009), but the nymphs initiated their activity 1–2 months earlier in the urban habitat (in both 2019 and 2021) compared to natural habitats. The most dramatic difference in this comparison was observed at the end of the tick season, because in natural habitats *H. concinna* adults and nymphs were only active until July (Hornok 2009), whereas in the cemetery until October/November. As *H. concinna* is a thermophilic tick species (Hubálek et al. 2003), the prolonged activity in 2019 might be explained by the commencing warm winter, and the later initiation of activity in 2020 by the same pre-seasonal influence of this unusual weather, similarly to the questing activity of *I. ricinus* as outlined above.

To the best of our knowledge, this is the first year-round seasonality assessment of *I. frontalis* in Central Europe. This tick species is known for its late autumn and winter activity under suboceanic climate in Western Europe (Agoulon et al. 2019), implying preference for cool weather, as also confirmed here under continental climate. This may be the main cause why in the spring of 2020 (following the mildest winter) *I. frontalis* was not found in the cemetery. In this study, most *I. frontalis* nymphs were collected late winter and early spring (February–April), whereas the peak activity of larvae was during late autumn (October–November), which is a pattern similar to the decline in nymphal activity from October until April next year, and early predominance of nymphs each year as reported in France under suboceanic climate (Agoulon et al. 2019).

Concerning morphotypes of *I. ricinus* recognized in this study, the majority of these variants were unique in the studied material, except for morphotype I which was repeatedly collected and showed the same distinguishing characters consistently. The gnathosoma of morphotype I (represented by nine females) was significantly different from the usual morphology of *I. ricinus* (e.g., as shown in Bugmyrin et al. 2016; Estrada-Peña et al. 2017), because the basis capituli was rectangular and palpal article III was medially curved. The most important trait of morphotype I, contrary to *I. ricinus*, was the flat shape of the anterior surface of basis capituli which was reported to be a distinguishing character between various *Ixodes* spp. (Arthur 1953; Hornok et al. 2017). However, molecular and phylogenetic analyses confirmed that all morphotypes observed during this study belong to *I. ricinus*.

In addition, morphological anomalies were noted in a minority of ticks, all belonging to *I. ricinus*. These morphological anomalies found among ticks collected in this study were different from those reported in a large-scale survey in Germany, because the latter mostly affected the legs of *I. ricinus* (Chitimia-Dobler et al. 2017). As a possible background factor promoting the occurrence of morphological anomalies in ticks of the present study, it should be taken into account that these ticks were collected in an urban area where the level of environmental contamination is relatively high. The chances for morphological abnormalities were reported to be higher in urban areas in case of *Dermacentor reticulatus* (Hornok et al. 2014), and also the rate of infection with tick-borne pathogens in association with polluted habitats (Alekseev and Dubinina 2008).

Previously, when *I. frontalis* specimens collected from birds in Hungary were molecularly analyzed (Hornok et al. 2016), the results clearly indicated for the first time the existence of two distinct genetic lineages (haplogroups A and B) within *I. frontalis* that are transported by birds in Central Europe. The separate clustering of these mitochondrial lineages was supported by high bootstrap values in both the *cox*1 and 16S rDNA phylogenetic analyses (Hornok et al. 2016), in line with the high rate of *cox*1 and 16S rDNA sequence divergence in this study. Importantly, the degree of *cox*1 sequence divergence between the two lineages (8.6% as shown here) exceeds the average *cox*1 sequence difference (6.1%) separating closely related ixodid species (Lv et al. 2014), although the 16S rRNA gene difference was lower (3.7%) due to the more conserved nature of this marker.

To the best of our knowledge, this is the first evidence on the sympatric occurrence of the two haplogroups of *I. frontalis* during questing in the same habitat. Based on the *cox*1 gene, haplogroup A was rather homogenous, with a single predominant haplotype which probably represented a local population. This is also confirmed by our finding that all nine molecularly analyzed larvae belonged to this mitochondrial lineage. By contrast, haplogroup B was heterogenous, as all 5 nymphs in this category belonged to different haplotypes. These were most likely introduced as larvae by birds from other locations. In line with this hypothesis, migratory birds (most importantly robins, *Erithacus rubecula*) were previously shown to carry nine *cox*1 haplotypes of haplogroup B when arriving in Hungary during the spring, and the majority of these were larvae (Hornok et al. 2016).

Considering the haplotypes of 25 *I. frontalis* specimens analyzed here, the majority belonged to haplogroup A, limiting the availability of nymphs from haplogroup B for morphological comparison. During the latter all important structures and parameters were examined which serve to recognize this species (Estrada-Peña et al. 2017), and which were regarded as relevant in separating nymphs of the relatively recently discovered and difficult-to-recognize new species *I. inopinatus* from those of the sibling species *I. ricinus* (Estrada-Peña et al. 2014: including scutal dimensions and the relative size of scutal and alloscutal setae). In conclusion, taking into account that in the nymph stage of *I. frontalis* there were no recognizable and consistent morphological differences between individuals belonging to either haplogroup A or B, but their *cox*1 sequence identities below 94% would indicate different species (Dantas-Torres 2018), we suggest that these data support their status as cryptic species. Nevertheless, morphological comparison of adult stages should ultimately confirm this.

## Supplementary Information

Below is the link to the electronic supplementary material.

Supplementary file 1 (PDF 2287 KB)

Supplementary file 2 (PDF 317 KB)

Supplementary file 3 (JPG 483 KB)

Supplementary file 4 (JPG 497 KB)

Supplementary file 5 (PDF 1144 KB)

Supplementary file 6 (PDF 2753 KB)

Supplementary file 7 (PDF 2350 KB)

Supplementary file 8 (PDF 2209 KB)

Supplementary file 9 (PDF 122 KB)

Supplementary file 10 (PDF 38 KB)

## Data Availability

The sequences obtained and/or analyzed during the current study are deposited in GenBank under the following accession numbers: OM108450-OM108465 for *I. ricinus* 16S rRNA gene, OM108447-OM108449 for *I. frontalis* 16S rRNA gene and OM108437-OM108443 for *I. frontalis cox*1 gene. All other relevant data are included in the manuscript and supplementary material or are available upon request by the corresponding author.
