# Supplementary figures and images for: Shift in the seasonality of ixodid ticks after a warm winter in an urban habitat with notes on morphotypes of Ixodes ricinus and data in support of cryptic species within Ixodes frontalis
Source: Exp Appl Acarol. 2022 Oct 25;88(1):127–38. doi: 10.1007/s10493-022-00756-1 (PMC9663398; doi:10.1007/s10493-022-00756-1)

**Supplementary Figure 1.**

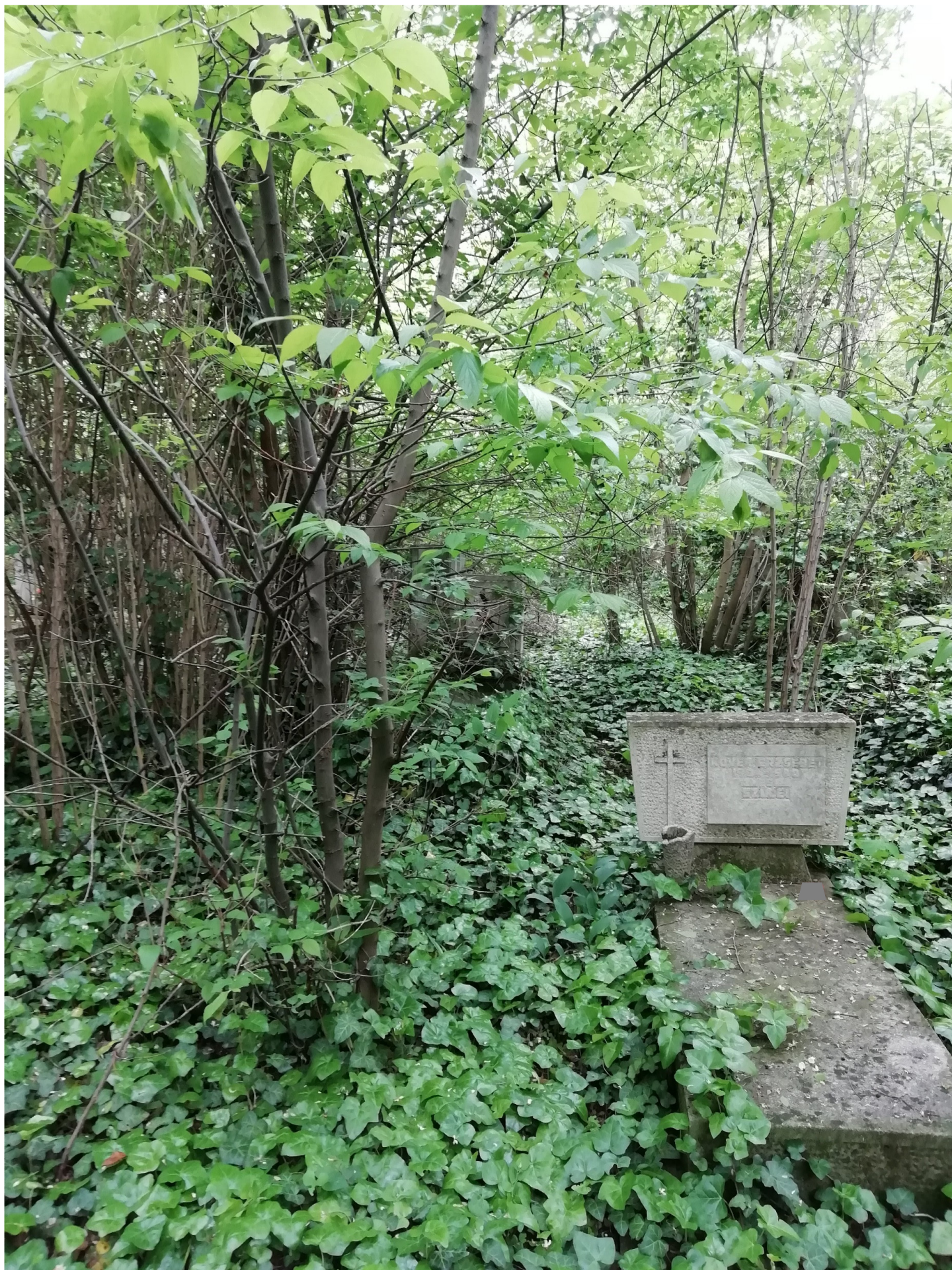

Supplement: Supplementary file 1 — (PDF 2287 KB) [file 10493_2022_756_MOESM1_ESM.pdf]

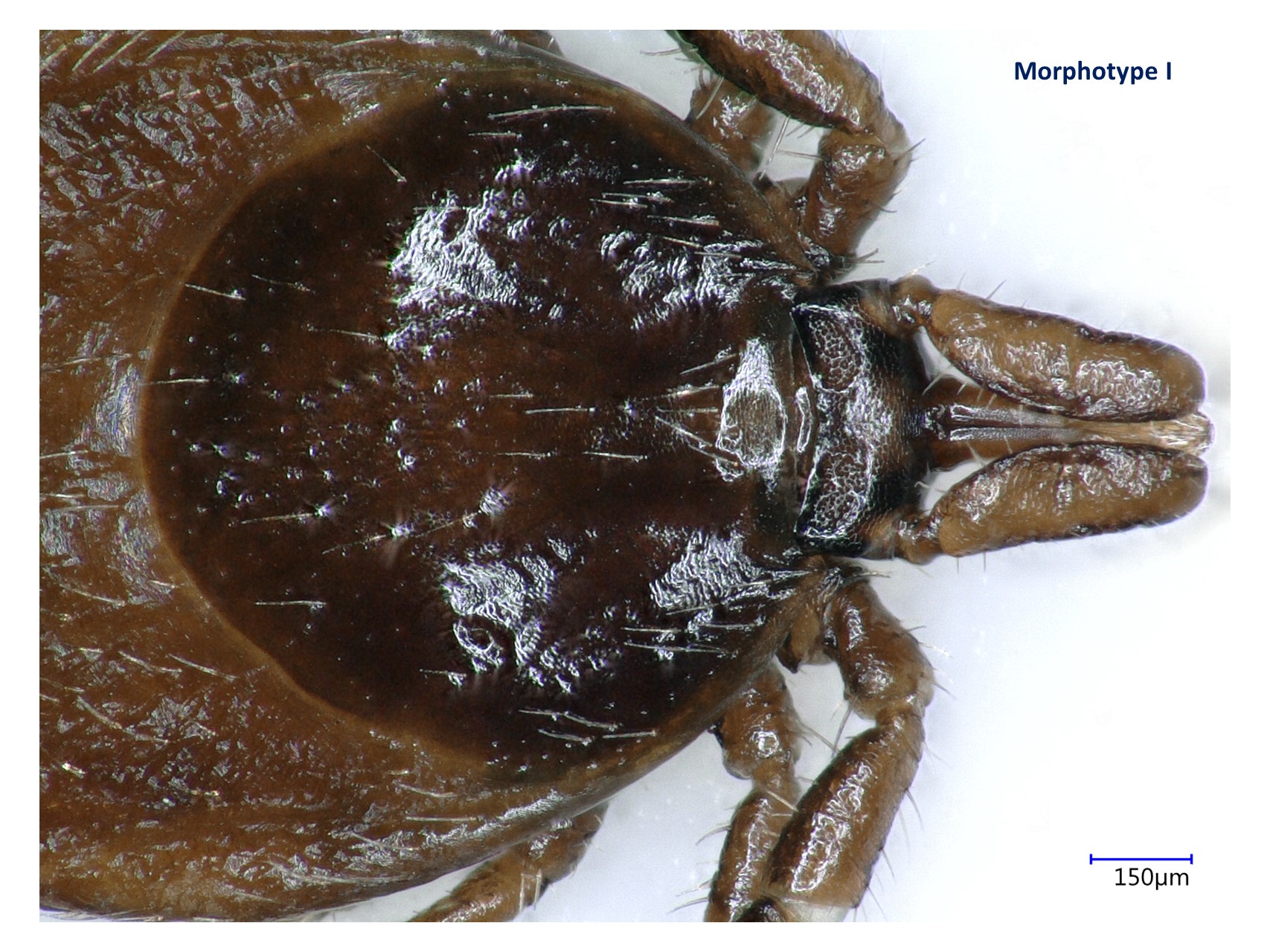

Supplement: Supplementary file 3 — (JPG 483 KB) [file 10493_2022_756_MOESM3_ESM.jpg]

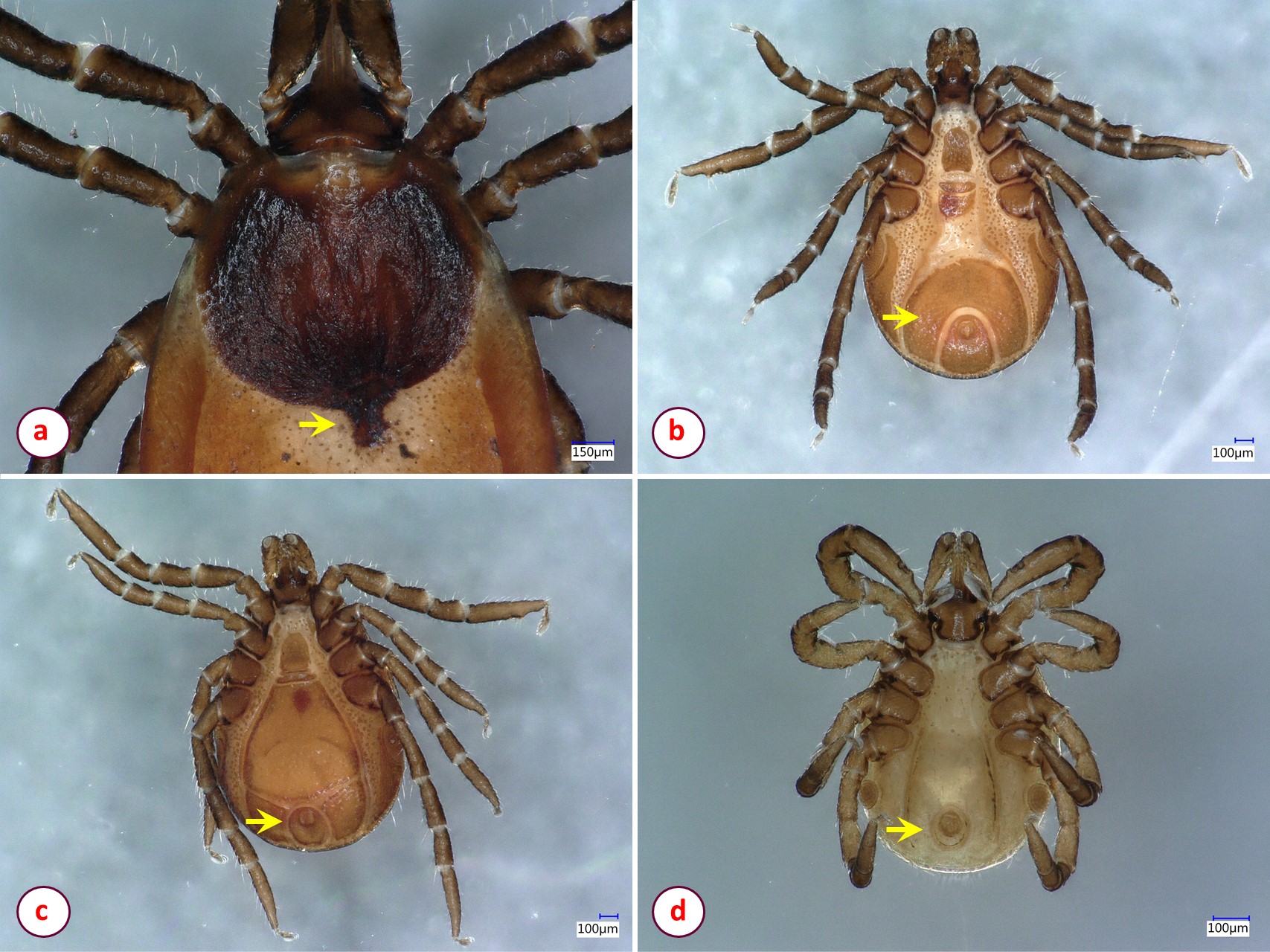

Supplement: Supplementary file 4 — (JPG 497 KB) [file 10493_2022_756_MOESM4_ESM.jpg]

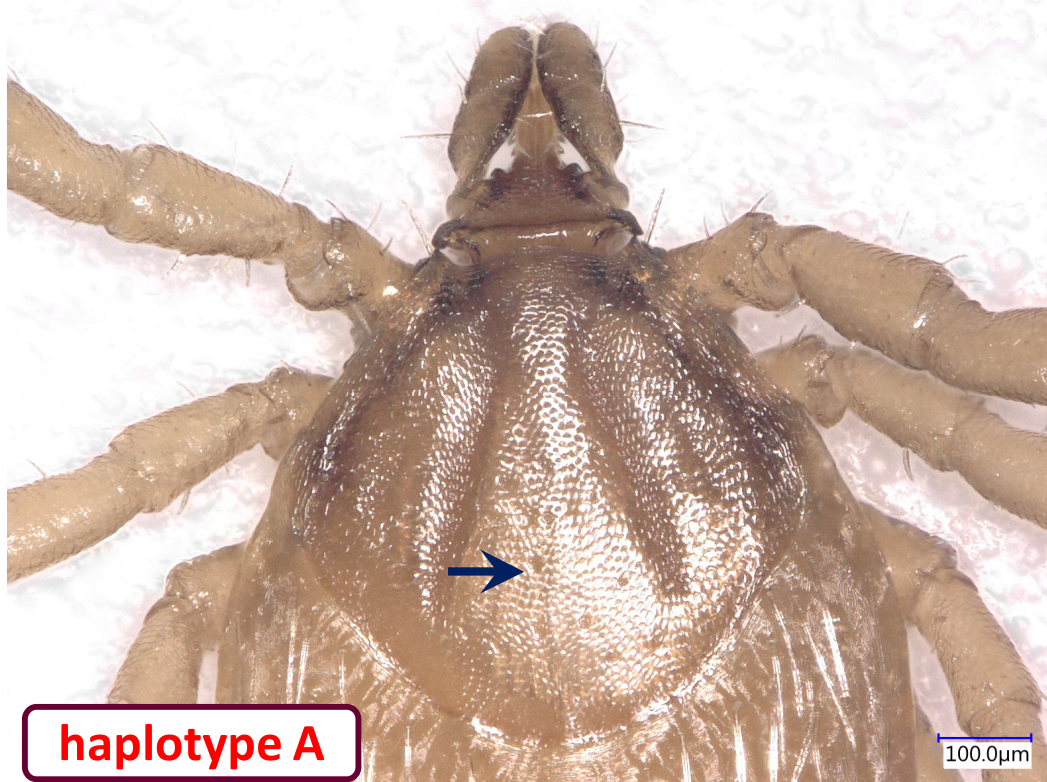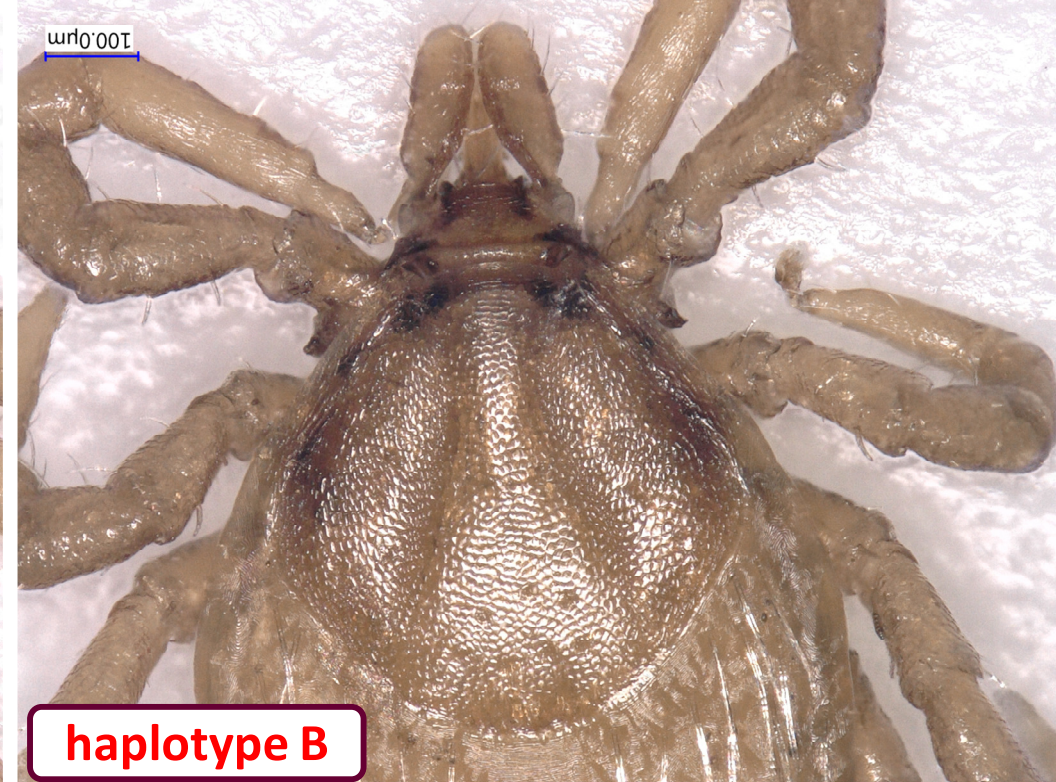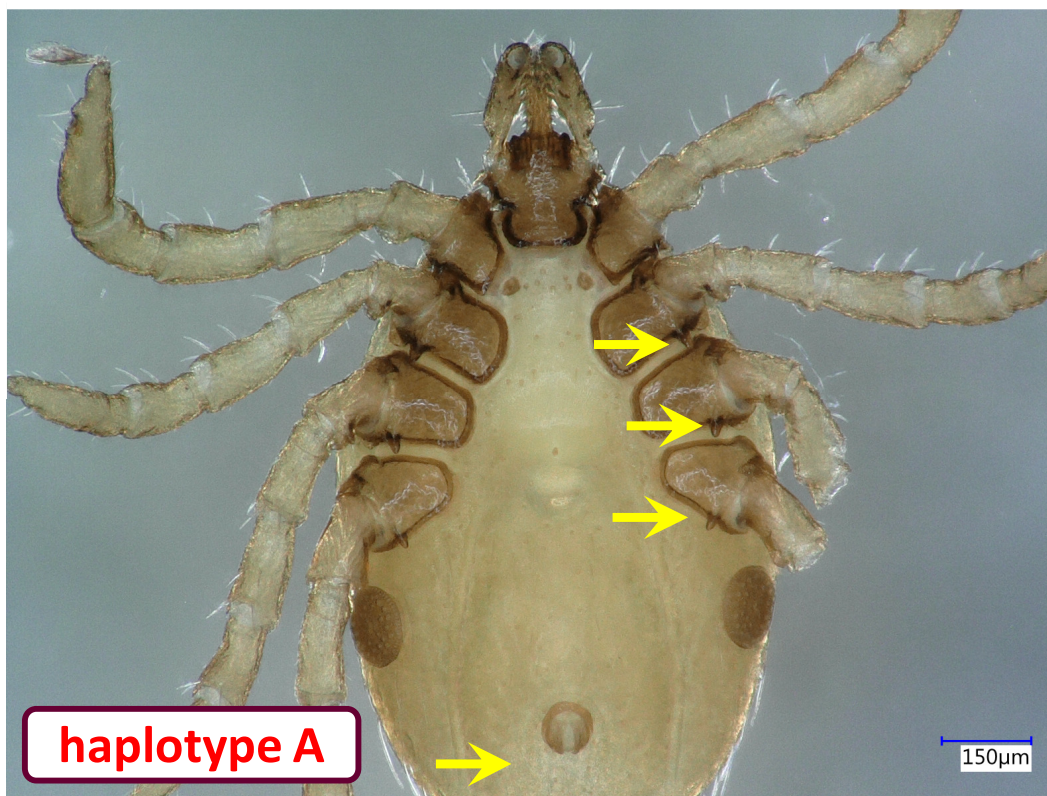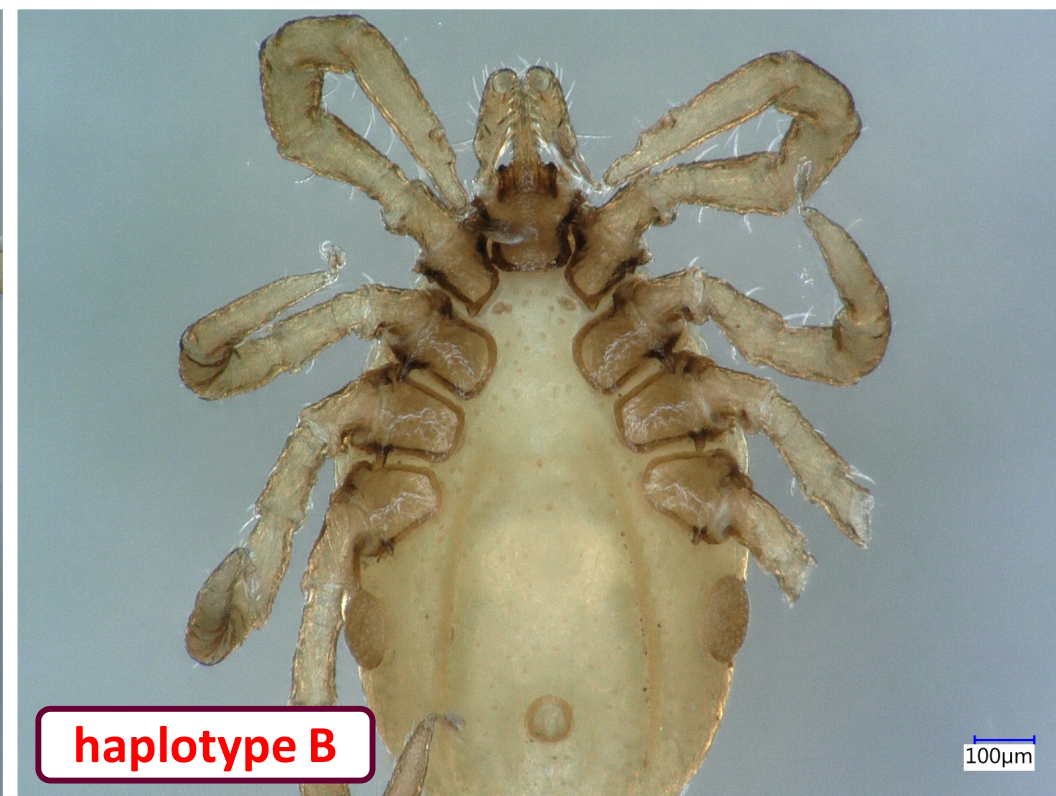

Supplement: Supplementary file 6 — (PDF 2753 KB) [file 10493_2022_756_MOESM6_ESM.pdf]

haplotype A

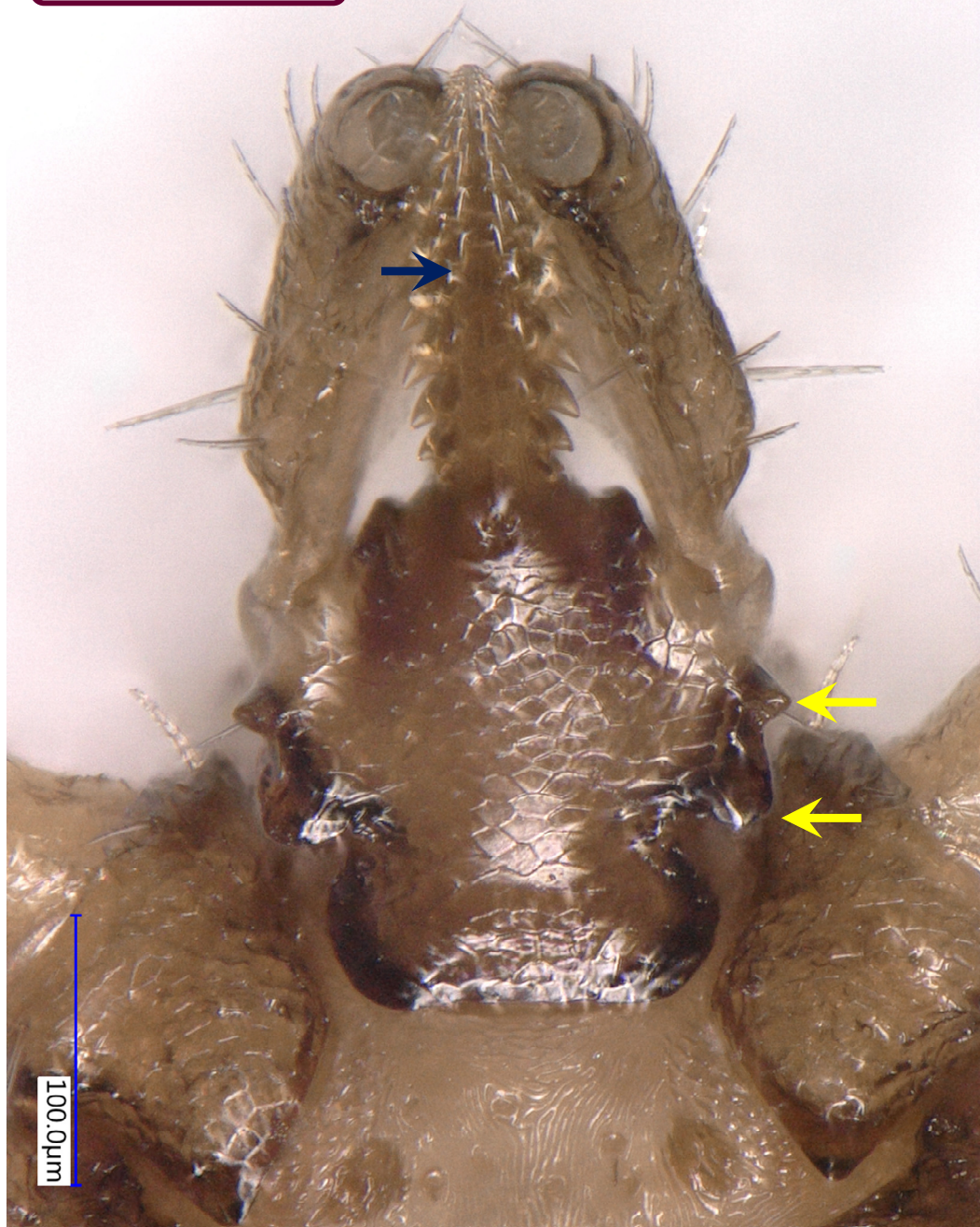

haplotype B

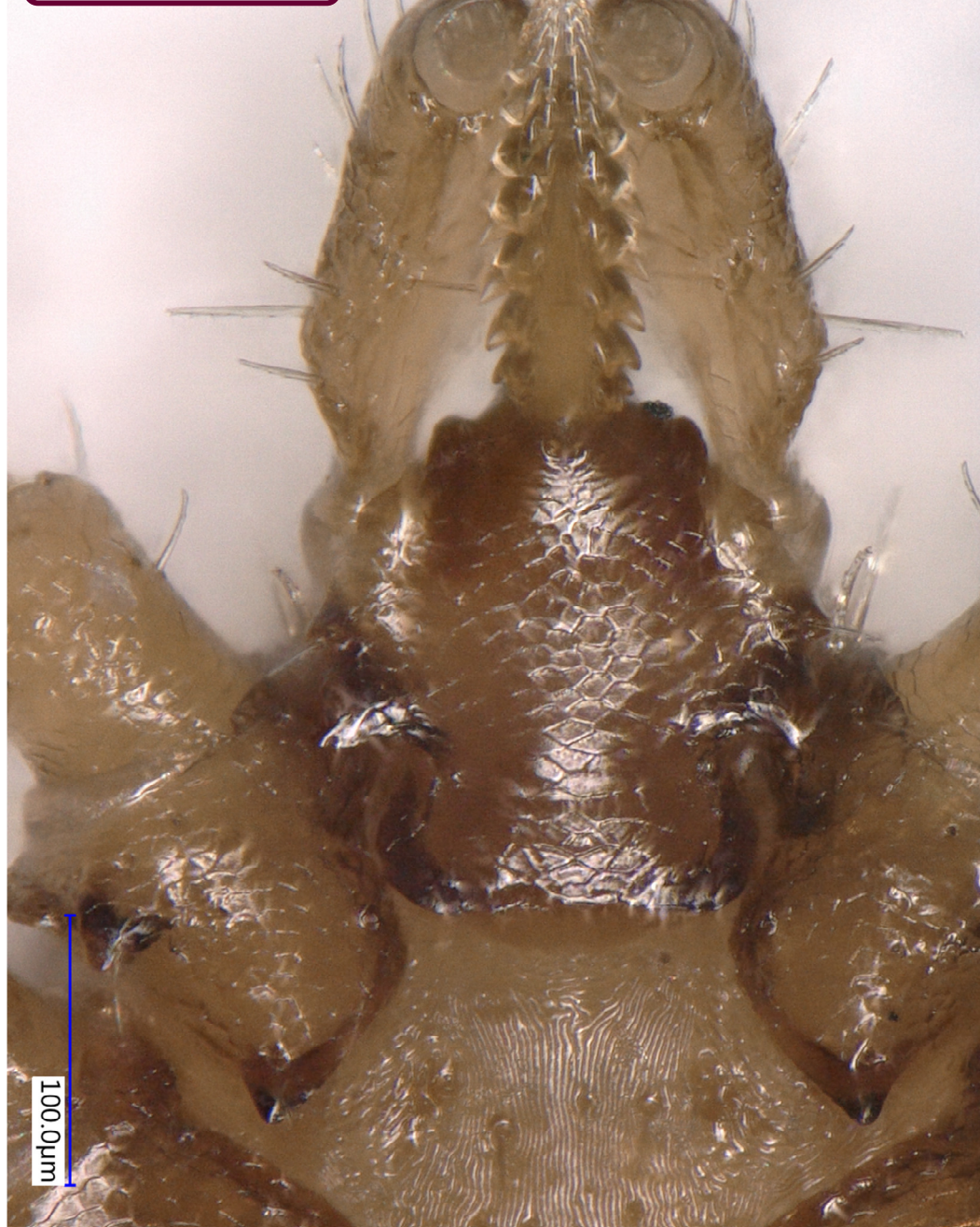

Supplement: Supplementary file 7 — (PDF 2350 KB) [file 10493_2022_756_MOESM7_ESM.pdf]

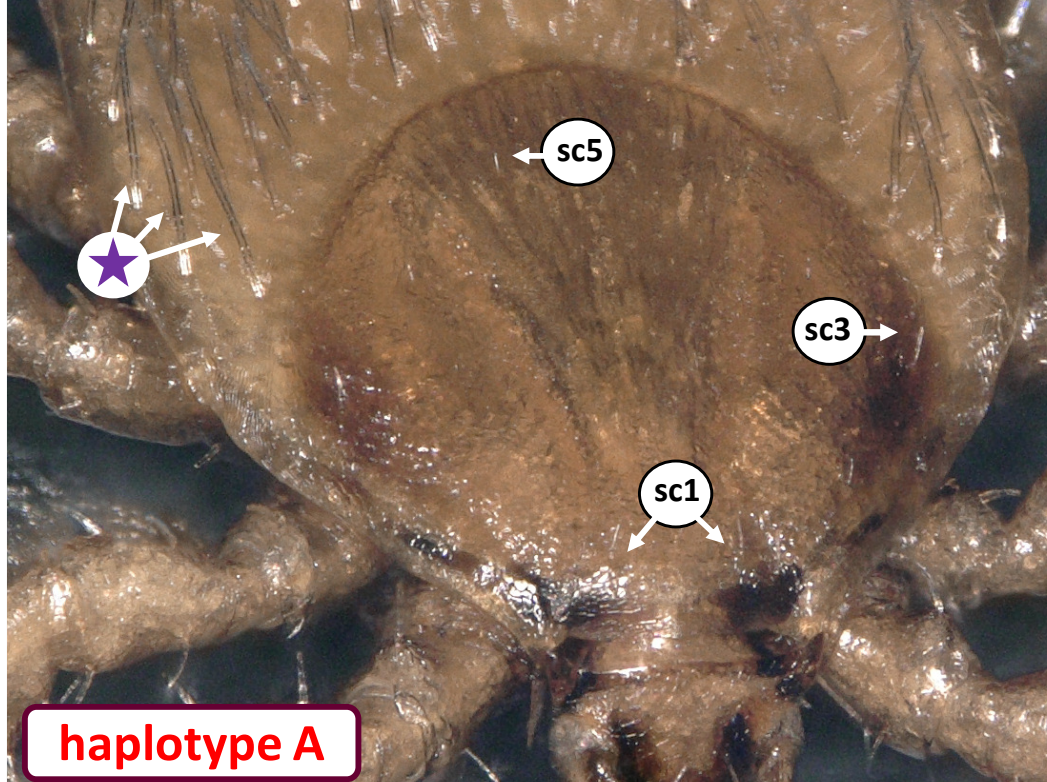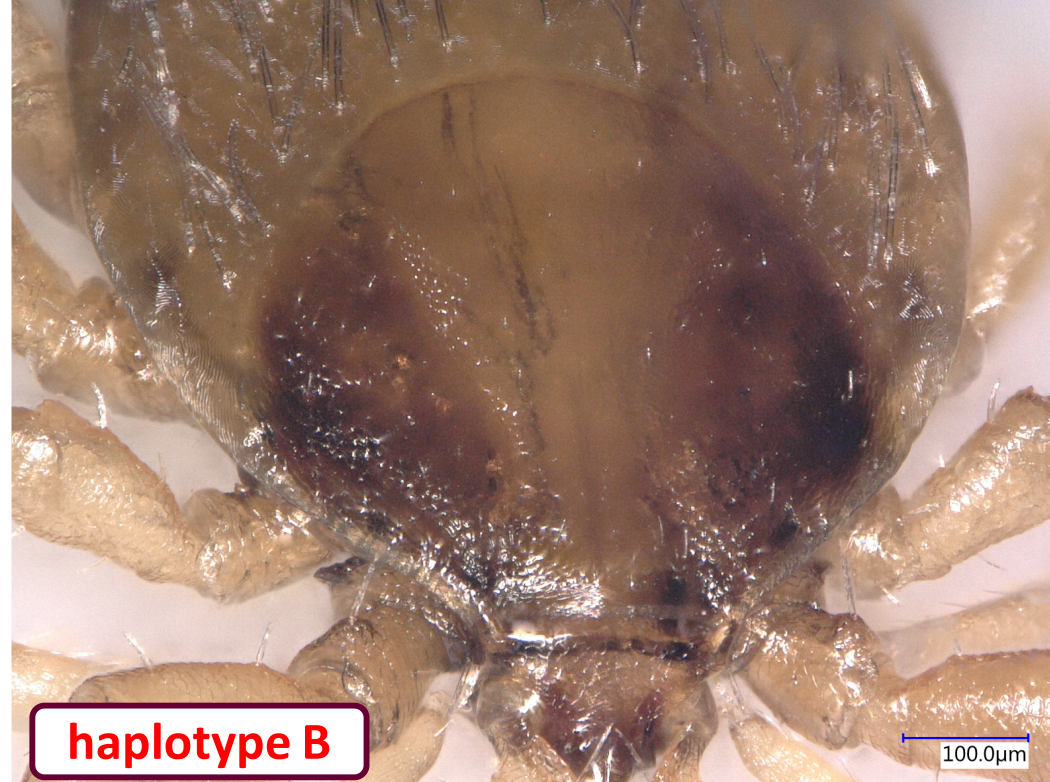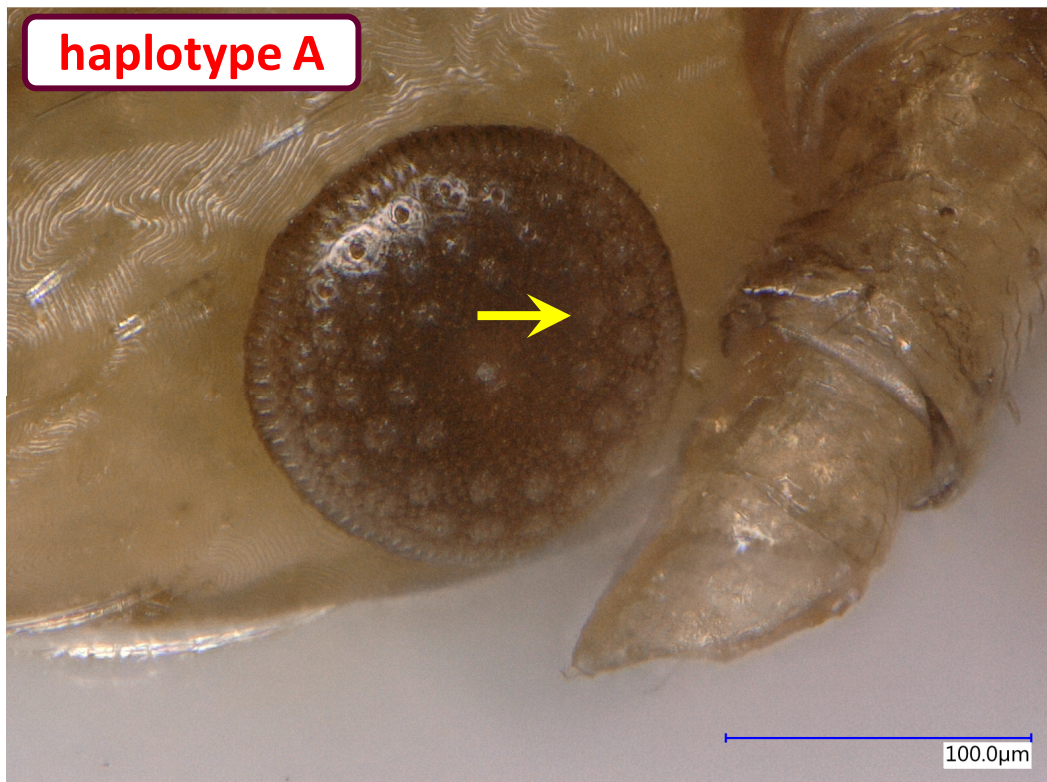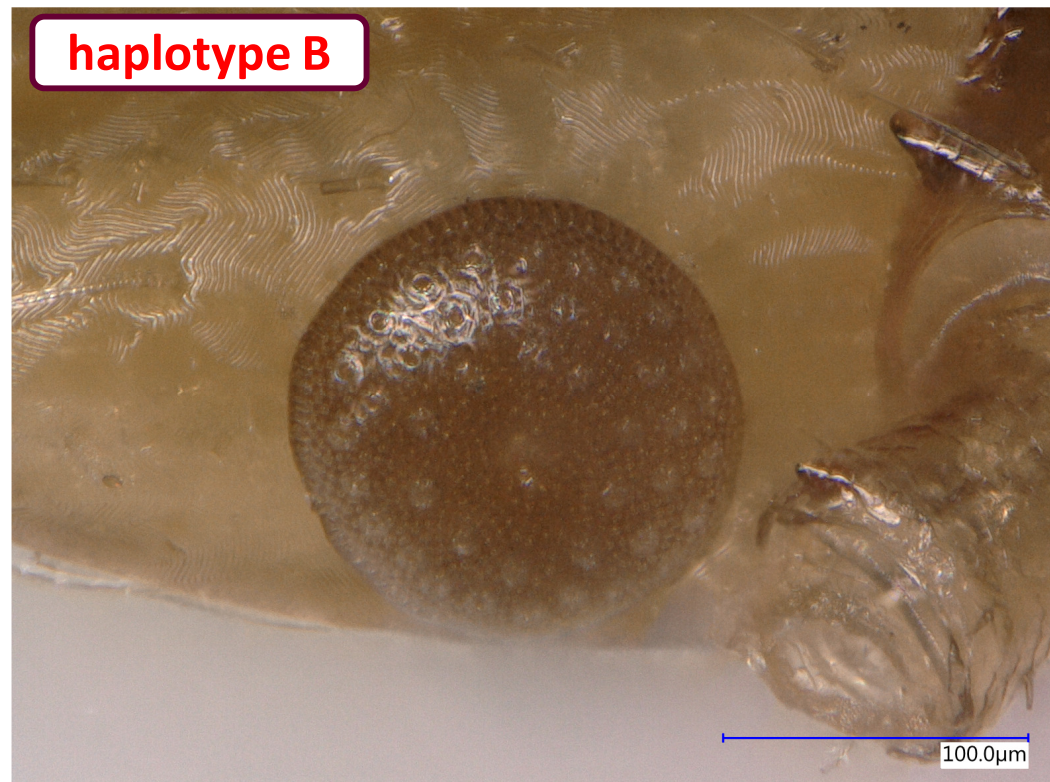

Supplement: Supplementary file 8 — (PDF 2209 KB) [file 10493_2022_756_MOESM8_ESM.pdf]
