# Supplementary material for: Shift in the seasonality of ixodid ticks after a warm winter in an urban habitat with notes on morphotypes of Ixodes ricinus and data in support of cryptic species within Ixodes frontalis
Source: Exp Appl Acarol. 2022 Oct 25;88(1):127–38. doi: 10.1007/s10493-022-00756-1 (PMC9663398; doi:10.1007/s10493-022-00756-1)
Supplement: Supplementary file 2 — (PDF 317 KB) [file 10493_2022_756_MOESM2_ESM.pdf]

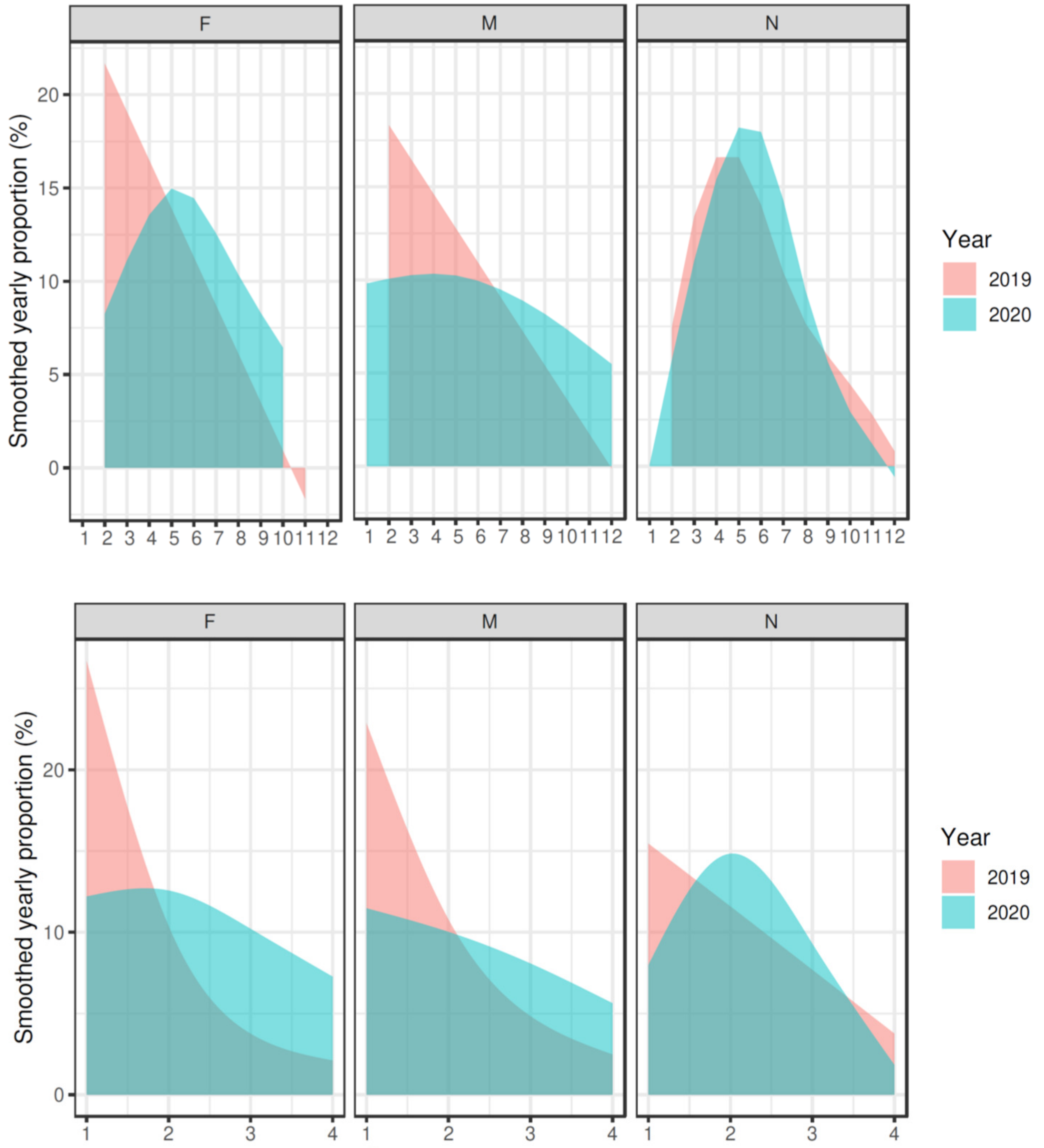

Smoothed yearly proportions of *Ixodes ricinus* adults (F, M) and nymphs (N) according to monthly or quarterly data in 2019 vs 2020.
