# Supplementary material for: Shift in the seasonality of ixodid ticks after a warm winter in an urban habitat with notes on morphotypes of Ixodes ricinus and data in support of cryptic species within Ixodes frontalis
Source: Exp Appl Acarol. 2022 Oct 25;88(1):127–38. doi: 10.1007/s10493-022-00756-1 (PMC9663398; doi:10.1007/s10493-022-00756-1)

Supplementary Figure 5. The phylogenetic relationships of morphotypes, malformed and typical specimens of *Ixodes ricinus*. A standard tick (specimen RIC16) was chosen on account of its 16S rRNA sequence identity with a tick collected in neighboring Slovakia (GU074590). The ticks used for the molecular-phylogenetic comparison included altogether nine ticks (six females, three males) showing "usual morphology" of *I. ricinus* (specimens RIC3, RIC7, RIC9, RIC11, RIC12, RIC13, RIC16, RIC17, RIC21). These had up to 6 bp sequence divergence from each other in the amplified part of their 16S rRNA gene. Specimens belonging to morphotype I (nine females: RIC4, RIC5, RIC6, RIC8, RIC10, RIC18, RIC19, RIC20, RIC24) showed up to 4 bp sequence divergence from each other and from the group showing "usual morphology". Morphotype II (a single female: RIC15) had 4 bp difference, whereas morphotype III (another female: RIC23) had only 1 bp difference from the standard tick (RIC16). Ticks showing morphological anomalies (I-IV.: RIC1, RIC22, RIC14, RIC2) had 1 bp, 1 bp, 5 bp, and 2 bp sequence divergence from the standard tick, respectively.

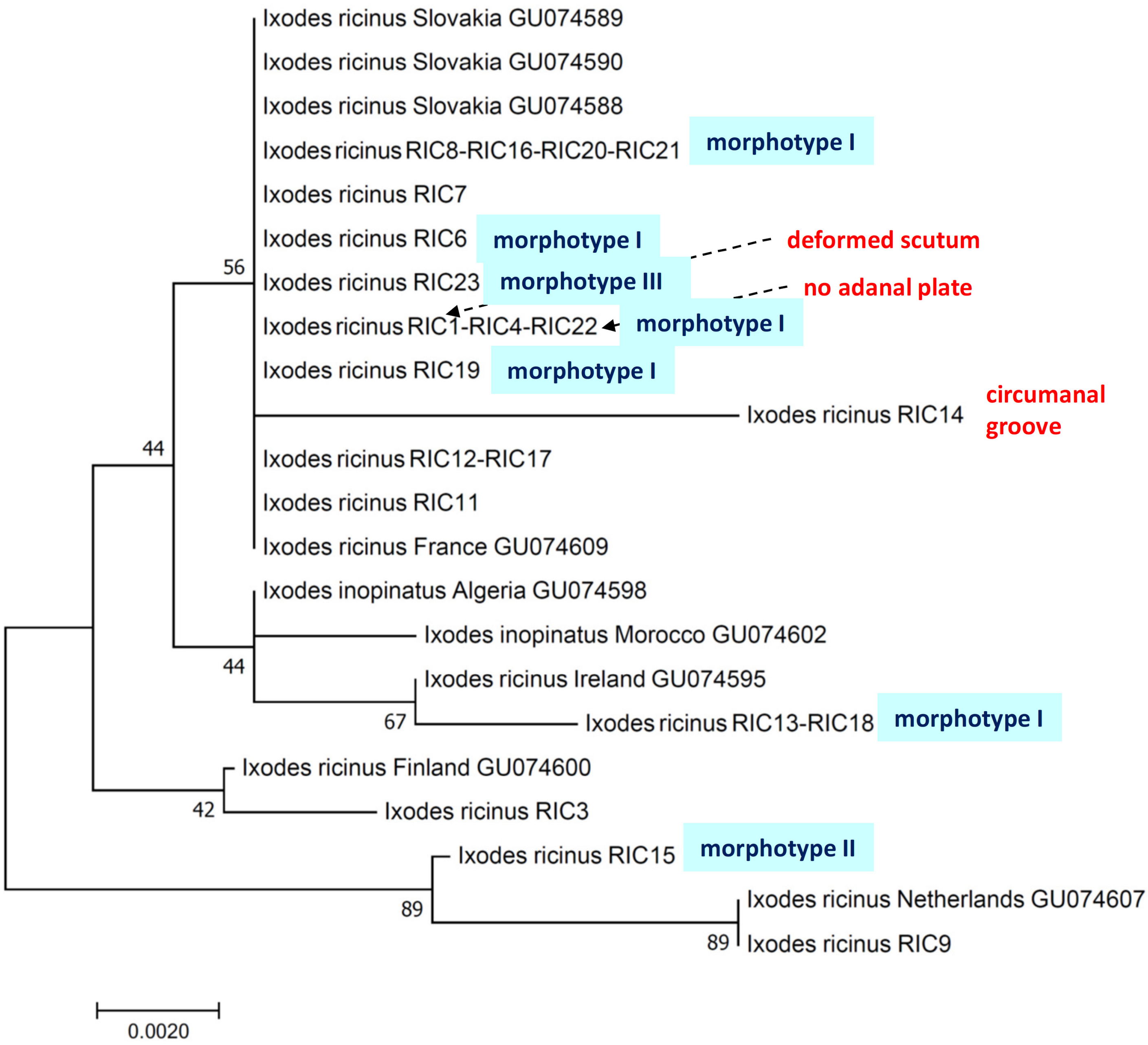

Supplement: Supplementary file 5 — (PDF 1144 KB) [file 10493_2022_756_MOESM5_ESM.pdf]
