# Supplementary material for: Shift in the seasonality of ixodid ticks after a warm winter in an urban habitat with notes on morphotypes of Ixodes ricinus and data in support of cryptic species within Ixodes frontalis
Source: Exp Appl Acarol. 2022 Oct 25;88(1):127–38. doi: 10.1007/s10493-022-00756-1 (PMC9663398; doi:10.1007/s10493-022-00756-1)
Supplement: Supplementary file 9 — (PDF 122 KB) [file 10493_2022_756_MOESM9_ESM.pdf]

	Ix. ricinus				*Ha. concinna*			
	**M**	**F**	**N**	**L**	**M**	**F**	**N**	**L**
JAN	–	–	–	–	–	–	–	–
FEB	9	11	4	–	–	–	–	–
MAR	51	44	143	–	–	–	3	–
APR	6	5	92	1	–	1	19	3
MAY	8	5	143	3	–	–	12	16
JUNE	6	5	88	–	–	1	39	45
JULY	5	9	41	1	1	–	28	2
AUG	3	2	42	1	–	–	23	2
SEPT	10	2	40	–	–	–	11	2
OCT	6	4	38	–	–	–	6	–
NOV	5	2	20	–	–	–	1	1
DEC	1	–	2	–	–	–	–	–
	**110**	**89**	**653**	**6**	**1**	**2**	**142**	**71**

percent:	*Ix. ricinus*				*Ha. concinna*			
	**M**	**F**	**N**	**L**	**M**	**F**	**N**	**L**
JAN	0	–	–	–	–	–	–	–
FEB	8.18	12.35	0.61	–	–	–	–	–
MAR	**46.36**	**49.43**	**21.89**	–	–	–	2.11	–
APR	5.45	5.61	14.08	16.67	–	50	13.38	4.22
MAY	7.27	5.61	**21.89**	50	–	–	8.45	22.53
JUNE	5.45	5.61	13.47	–	–	50	**27.46**	**63.38**
JULY	4.54	**10.11**	6.27	16.67	100	–	19.71	2.81
AUG	2.72	2.24	6.43	16.67	–	–	16.19	2.81
SEPT	**9.09**	2.24	6.12	–	–	–	7.74	2.81
OCT	5.45	4.49	5.81	–	–	–	4.22	–
NOV	4.54	2.24	3.06	–	–	–	0.7	1.4
DEC	0.9	–	0.3	–	–	–	–	–
	**99.95**	**99.93**	**99.93**	**100.01**	**100**	**100**	**99.96**	**99.96**

Tick abundance - 2019

	Ix. ricinus				*Ha. concinna*			
	**M**	**F**	**N**	**L**	**M**	**F**	**N**	**L**
JAN	2	–	1	–	–	–	–	–
FEB	6	3	11	–	–	–	–	–
MAR	12	6	87	–	–	–	–	–
APR	3	4	41	–	–	–	–	–
MAY	19	15	130	1	5	3	24	–
JUNE	5	9	136	7	–	1	47	116
JULY	7	4	75	35	1	1	36	4
AUG	1	4	20	23	1	–	37	13
SEPT	15	4	19	4	–	–	15	2
OCT	2	4	22	12	–	–	4	2
NOV	–	–	–	–	–	–	–	–
DEC	1	–	1	–	–	–	–	–
	**73**	**53**	**543**	**82**	**7**	**5**	**163**	**137**

percent:	*Ix. ricinus*				*Ha. concinna*			
	**M**	**F**	**N**	**L**	**M**	**F**	**N**	**L**
JAN	2.73	–	0.18	–	–	–	–	–
FEB	8.21	5.66	2.02	–	–	–	–	–
MAR	16.43	11.32	16.02	–	–	–	–	–
APR	4.1	7.54	7.55	–	–	–	–	–
MAY	**26.02**	**28.3**	23.94	1.21	71.42	**60**	14.72	–
JUNE	6.84	16.98	**25.04**	8.53	–	20	**28.83**	**84.67**
JULY	9.58	7.54	13.81	42.68	14.28	20	22.08	2.91
AUG	1.36	7.54	3.68	28.04	14.28	–	22.69	9.48
SEPT	**20.54**	7.54	3.49	4.87	–	–	9.2	1.45
OCT	2.73	7.54	4.05	14.63	–	–	2.45	1.45
NOV	–	–	–	–	–	–	–	–
DEC	1.36	–	0.18	–	–	–	–	–
	**99.9**	**99.96**	**99.96**	**99.96**	**99.98**	**100**	**99.97**	**99.96**

Tick abundance - 2020

	Ix. ricinus				*Ha. concinna*			
	**M**	**F**	**N**	**L**	**M**	**F**	**N**	**L**
JAN	1	–	–	–	–	–	–	–
FEB	14	27	34	–	–	–	–	–
MAR	57	38	162	22	–	–	–	–
APR	61	36	282	8	2	–	15	1
MAY	39	37	260	86	13	7	46	84
	**172**	**138**	**738**	**116**	**15**	**7**	**61**	**85**

percent:	*Ix. ricinus*				*Ha. concinna*			
	**M**	**F**	**N**	**L**	**M**	**F**	**N**	**L**
JAN	0.58	–	–	–	–	–	–	–
FEB	8.13	19.56	4.6	–	–	–	–	–
MAR	33.13	**27.53**	21.95	18.96	–	–	–	–
APR	**35.46**	26.08	**38.21**	6.89	13.33	–	24.59	1.17
MAY	22.67	26.81	35.23	74.13	86.67	100	**75.4**	**98.82**
	**99.97**	**99.98**	**99.99**	**99.98**	**100**	**100**	**99.99**	**99.99**

Tick abundance - 2021 winter, spring
