# Supplementary material for: Shift in the seasonality of ixodid ticks after a warm winter in an urban habitat with notes on morphotypes of Ixodes ricinus and data in support of cryptic species within Ixodes frontalis
Source: Exp Appl Acarol. 2022 Oct 25;88(1):127–38. doi: 10.1007/s10493-022-00756-1 (PMC9663398; doi:10.1007/s10493-022-00756-1)
Supplement: Supplementary file 10 — (PDF 38 KB) [file 10493_2022_756_MOESM10_ESM.pdf]

Supplementary Table 2. Haplotypes of selected *Ixodes frontalis* specimens according to their month of collection and developmental stage.

	Haplogroup A		Haplogroup B		
	Larvae	Nymphs	Larvae	Nymphs	Female
February	NA	4	NA	2	NA
March	2	5	-	1	1
April	ND	-	ND	1	NA
September	NA	1	NA	1	NA
October	5	NA	-	NA	NA
November	2	NA	-	NA	NA

Abbreviations: ND - not done, NA - not available
